# Effect of Lavender (*Lavandula angustifolia* L.) syrup on olfactory dysfunction in COVID-19 infection: A pilot controlled clinical trial

**DOI:** 10.22038/AJP.2021.18420

**Published:** 2022

**Authors:** Fataneh Hashem-Dabaghian, Sadegh Ali Azimi, Mohsen Bahrami, Seied-Amirhossein Latifi, Ayesheh Enayati, Marzieh Qaraaty

**Affiliations:** 1 *Research Institute for Islamic and Complementary Medicine, School of Persian Medicine, Iran University of Medical Sciences, Tehran, Iran*; 2 *Infectious Diseases Research Center, Golestan University of Medical Sciences, Gorgan, Iran*; 3 *Researcher of Persian Medicine, Tehran, Iran *; 4 *Traditional and Complementary Medicine Research Center,* *Faculty of Medicine, Arak University of Medical Sciences, Arak, Iran*; 5 *Ischemic research Center, Golestan University of Medical Sciences, Gorgan, Iran*; 6 *Clinical Research Development Unit (CRDU), Sayad Shirazi Hospital, Golestan University of Medical Sciences, Gorgan, Iran *

**Keywords:** COVID-19, Anosmia, Herbal medicine, Lavendula, Persian medicine

## Abstract

**Objective:**

The effect of lavender syrup on COVID-19-induced olfactory dysfunction** (**OD**)** has been assessed in this study.

**Materials and Methods:**

This pilot clinical trial was conducted in Gonbad-E-Kavoos (Golestan province, Iran). Twenty-three outpatients with COVID-19 and OD in lavender group took 9 ml of lavender syrup/bid for 3 weeks along with the standard COVID-19 treatments and 20 patients in control group took only standard COVID-19 treatments. The severity of OD was assessed by the visual analogue scale (VAS). Data analysis was performed by Friedman and Mann-Whitney tests using SPSS software.

**Results:**

The mean± standard deviation of age was 36.6±9.1, and 42.6±10.4 years (p=0.05), and the duration of symptoms was 7.4±3.5, and 7.5±3.4 days (p=0.98) in the lavender and control group, respectively. The VAS score for OD decreased from 6.8±3.04 to 0.26±0.86 in the lavender group and from 5.3±3.4 to 1±2.61 in the control group. Although, VAS for OD was significantly decreased in both groups (p<0.001), the amount of VAS decrease was 6.6±2.9 scores in the lavender group, and 4.3±4 in the control group (p=0.03). No side effects were observed in the lavender group.

**Conclusion:**

The present study showed that lavender syrup is an effective treatment for COVID-19-induced OD. It is suggested to conduct further studies with larger sample size.

## Introduction

Olfactory dysfunction (OD) is defined by reduced or distorted ability to smell during sniffing (ortho-nasal olfaction) or eating (retro-nasal olfaction) (Spinato et al., 2020 ▶); it is a common symptom in Coronavirus disease 2019 (COVID-19) infection (Huang et al., 2019 ▶) with the overall prevalence of 31-67% (Borsetto et al., 2020 ▶). 

OD is not a life-threatening problem, but has a severe impact on the quality of life, and may cause depression and anxiety if it is chronic (Boesveldt et al., 2017 ▶). 

Severe Acute Respiratory Syndrome-Coronavirus-2 (SARS-COV-2)-related OD may be a COVID-19 specific syndrome (Han et al., 2020 ▶), which is often without nasal obstruction, rhinorrhea, or congestion (Gengler et al., 2020 ▶). 

The etiology and mechanism of OD in COVID-19 are not clearly known. It may occur as a result of peripheral injury the peripheral injury of the olfactory nerve and branches and olfactory epithelium supporting cells (Han et al., 2020 ▶; Laurendon et al., 2020 ▶). 

There is no specific treatment for COVID-19-induced OD and the efficacy of available treatments for patients with COVID-19-related OD is unknown (Whitcroft and Hummel, 2020 ▶). 

*Lavendula angustifolia* (lavender) is a plant from the Lamiaceae family (Cavanagh and Wilkinson, 2002 ▶) with a wide range of activities such as antibacterial, antifungal, antioxidant, anti-inflammatory (Cavanagh and Wilkinson, 2002 ▶; DA Silva et al., 2015 ▶), and anti-viral effect (DA Silva et al., 2020 ▶). In addition, lavender has been shown to be clinically effective in patients with anxiety, sleep disorders, headache, convulsion, cognitive disorders, and depression (Koulivand et al., 2013 ▶). 

In Persian medicine (PM) literature, lavender is named “*Ostokhoddus*”. It has been mentioned that the plant is effective for strengthening the body, brain, heart, memory and mood, and is especially very important for its cleansing effect in the body and the brain (Amin et al., 2019 ▶). 

This study was conducted to assess the effects of lavender syrup in outpatients with COVID-19- induced OD. 

## Materials and Methods

This pilot clinical trial was a part of the study for evaluating the effects of lavender syrup on COVID-19 signs and symptoms, which was conducted in 2020 in the clinic of the Gonbad-E-Kavoos Health center, Golestan University of Medical Sciences, Iran (registration code: IRCT20110907007511N4).

**Preparation of drug**

Here, 100 g of dried aerial parts of lavender (*Lavandula angustifolia* L.) was washed, then macerated in 1000 ml of water for 3 hr. In the next step, it was boiled for 10 min and concentrated. Then, 5 g of the obtained extract was brought to 100 g with 66.5 g of honey and 28.5 g of water. The resulting syrup was poured into 120 ml sterile jars, sealed and sterilized by autoclave (Aghili Khorasani, 2012 ▶; Fleming, 2009 ▶).

**Participants**

Polymerase chain reaction (PCR) positive or highly suspicious COVID-19 patients (based on the sixth edition of the flowchart of diagnosis and treatment of COVID-19) (Ministry of health, 2020) were included in our study. The inclusion criteria were being 18-65 years old, having fever or flushing along with at least one of the following symptoms: dry cough, respiratory rate ≥24 breaths per minute, malaise, fatigue, headache, sore throat, OD, gustatory dysfunction, chest pain, diarrhea and vomiting. 

The exclusion criteria were smoking, pregnancy, breastfeeding, having underlying diseases (heart, lung, and liver or kidney diseases) or a known allergy to lavender. Drug adherence less than 80%, worsening of the symptoms and need for hospitalization were the attrition criteria. 

**Study design**

After confirming the eligibility and filling the informed consent form, the patients were divided into two groups through block randomization. 

**Interventions**

Both groups received the standard treatment for COVID-19 based on the sixth national guideline (Ministry of health, 2020). Complementary interventions in lavender group was 9 ml lavender syrup twice a day for 3 weeks. 

**Outcomes**

To define the severity of symptoms, patients were followed by telephone once a week for up to 3 weeks and then, 2 and 4 weeks after discontinuing the intervention. The severity of OD was assessed by an 11-point visual analogue scale (VAS). Participants were asked to choose a value of 0-10 on the VAS scale based on their perception of OD severity (Doulaptsi et al., 2018 ▶).

**Statistical analysis**

The data were analyzed by SPSS software (Version 17). The mean± standard deviation, and frequency percentage were employed for describing variables. Friedman and U Mann- Whitney tests were used to analyze the data. A P value less than 0.05 was considered significant. 

## Results

**Figure 1:**
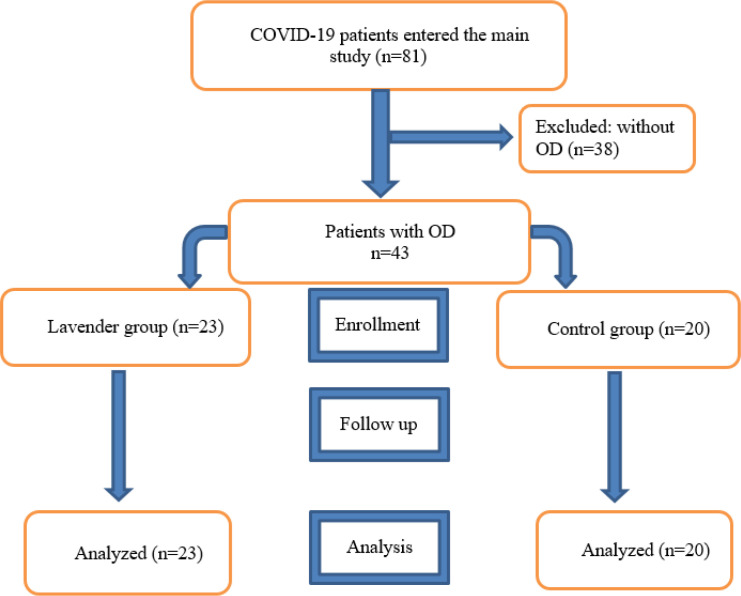
The CONSORT flow diagram of the study

Twenty-three patients with COVID-19-induced OD in the lavender group and 20 patients in the control group completed the study and data were analyzed. The CONSORT flow diagram is shown in Figure 1. 

Table 1 presents the baseline characteristics of the participants. Ten patients (23.25%) reported that OD was their first symptom. Rhinorrhea or nasal obstruction was reported in 6 (13.95%) patients. 

**Figure 2:**
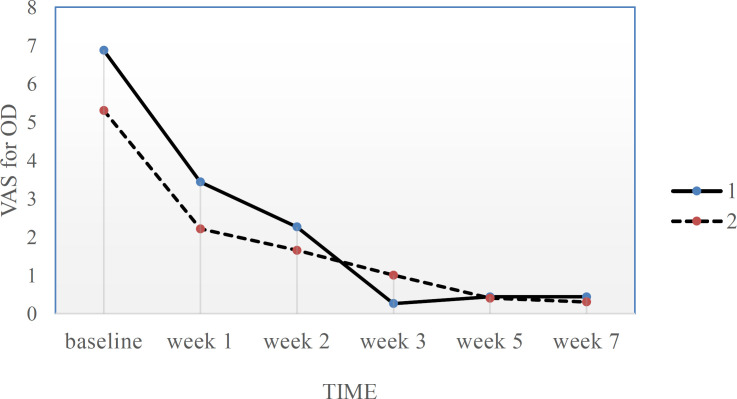
OD severity at 6 time points. 1=Lavender group, 2=control group, VAS for OD: the severity of olfactory dysfunction

VAS score for OD severity during the study is presented in Table 2 and Figure 2. 

According to Table 2, the severity of OD decreased in both groups after 3 weeks and after discontinuing the intervention (P<0.001); but the amount of olfactory improvement after 3 weeks was significantly larger in the lavender group than the control group (p=0.036). 

OD was completely improved (VAS=0) in 21 (91.3%) patients of the lavender group, and 16 (80%) patients in the control group (p=0.261) after 3 weeks of the study. Four weeks after discontinuing the intervention, 22 (95.6%) members of the lavender group and 19 (95%) members of the control group did not show any degree of OD (p=0.364). 

There was no relationship between the age and sex, with the baseline severity of OD in this study (p=0.196 and 0.454, respectively) as assessed by spearman’s rho test. 

There was also no significant relationship between age (p=0.206), sex (p=0.678), and the baseline severity of OD (p=0.766) with complete recovery of OD after third weeks.

Thirty-nine out of 43 participants (90.6%) had gustatory dysfunction at the baseline [21 participants in the lavender group, and 18 participants in the control group (p=0.558)]. After 3 weeks, VAS score of gustatory dysfunction was decreased from 6.04±3.18 to 0 in the lavender group (p<0.001), and was decreased from 4.5±3.41 to 0.61±1.91 in the control group (p<0.001). Although, both groups experienced a significant decrease in gustatory dysfunction, but the comparison between groups was not statistically significant. The amount of VAS decrease for gustatory dysfunction (VAS3-VAS1) was 5.5±3.5 in the lavender group, and was 3.5±4.07 in the control group (p=0.082). 

No side effect was observed in the lavender group.

**Table 1:** The basement characteristics of participants

Variables	Lavender group (n=23)	Control group (n=20)	p value
Age (year) (mean±SD)	36.6±9.13	42.65±10.4	0.053
Sex - n (%)	male: 17 (73.9)	male:7 (35)	0.015
	female: 6 (26.1)	Female: 13 (65)	
BMI (mean±SD)	25.33±4.09	27.64±4.56	0.088
History of URT disorders	Allergic rhinitis:1 Sinusitis:2 Polyp:1	Allergic rhinitis:1	0.423
Timing of OD onset (day) (mean±SD)	7.43±3.57	7.45±3.44	0.989
PCR positive - n (%)	20 (86.9)	18 (0.9)	0.299
Severity of OD - n (%)	Mild: 4 (17.4)	9 (45)	0.145
	Moderate: 7 (30.4)	4 (20)	
	Severe: 12 (52.2)	7 (35)	
Temperature (°C) (mean±SD)	36.58±0.3	36.51±0.15	0.452
Pulse rate(beat/min) (mean±SD)	86.34±11.32	87.9±13.78	0.591
Respiratory rate (number/minute) (mean±SD)	17.69±0.7	17.9±0.44	0.22
O_2_ saturation (percent) (mean±SD)	97.69±1.66	97.7±1.68	0.889

SD: standard deviation,

°C: degree of Celsius, URT: upper respiratory tract

**Table 2:** VAS score for OD during the study

	Lavender group	Control group	p value⃰
Baseline	6.86±3.04	5.3±3.45	0.11
Week 1	3.43±3.67	2.21±2.43	0.413
Week 2	2.26±3.55	1.65±3.24	0.364
Week 3	0.26±0.86	1±3.24	0.293
Changes^α^ from baseline to week 3	6.6±2.93	4.3±4.07	0.036
p value**	<0.001	<0.001	
Week 2^ ɣ^	0.43±2.08	0.4±1.78	0.947
Week 4^ ɣ^	0.43±2.08	0.3±1.34	0.947
Changes^β^ from baseline to month 1 ^ɣ^	6.43±3.24	5±3.27	0.136
p value**	<0.001	<0.001	

*: U Mann-Whitney test, **: Friedman test, ^ɣ^: after discontinuing the intervention, α: VAS at 3^rd^ week–VAS at baseline, β: VAS at 1^st^ month–VAS at baseline.

## Discussion

This study showed the effect of lavender syrup on OD in COVID-19-infected patients. Although both groups experienced improvement of OD during the study, the lavender group showed a greater benefit due to the intervention compare to the control group. 

Of the participants, 53% experienced OD, which is consistent with the existing data (Borsetto et al., 2020 ▶). 

Klopfeistein et al. reported that anosmia began 4.4 (±1.9 [1–8]) days after infection onset, and 98% of patients recovered within 28 days (Klopfenstein et al., 2020). 

Anosmia without nasal obstruction is a highly specific indicator of COVID-19 (Gengler et al., 2020 ▶). In the present study, only 6 patients (13.95%) had rhinorrhea or nasal obstruction. 

Although the exact pathophysiology of COVID-19- induced OD is unclear, it is probably the consequence of the inflammation of non-neuronal cell types such as olfactory epithelium supporting cells, microvilli cells, Bowman’s gland cells, horizontal basal cells and olfactory bulb periocytes. The long-lasting OD after COVID-19 infection might be due to the involvement of stem cells. Disruption of cells in the olfactory neuro epithelium may result in inflammatory changes; it causes subsequent olfactory neuron damage and impairs subsequent neurogenesis (Goncalves and Goldstein, 2016 ▶; Brann et al., 2020 ▶). 

There are no shreds of evidence to show the effect of lavender on OD. Although, the neuroprotective properties of lavender has been proven (Koulivand et al., 2013 ▶), the mechanism of action of Lavender in OD has not been clearly known. This plant has some constituents such as polyphenols, and esters, which are known for their anti-inflammatory properties (Cavanagh and Wilkinson, 2002 ▶; Basch et al., 2004 ▶; Hajhashemia et al., 2003 ▶). Therefore, lavender may be effective on OD by its anti-inflammatory activities.

The angiotensin-converting-enzyme 2 receptor, which is required for SARS-CoV-2 entry is highly expressed on the olfactory epithelium (Sungnak et al., 2020 ▶). Some plant flavonoids have shown to have angiotensin converting enzyme inhibitory effect (Nileeka Balasuriya and Vasantha Rupasinghe, 2011 ▶). The effect of lavender-derived flavonoids on the angiotensin-converting-enzyme 2 receptor, and this probable mechanism for treatment of COVID-19-OD should be evaluated. 

One study has shown the *in vitro* antiviral effect of essential oil of *L. angustifolia* against influenza type A (H1N1) (Vimalanathan and Hudson, 2014 ▶). The effect of lavender on COVID-19 virus should be evaluated in future studies. 

The available data suggest that short-term therapy with lavender is relatively safe. It should be used cautiously in patients with known allergy to lavender and should be avoided during pregnancy and breastfeeding. Skin irritation and headache were reported after local use of lavender oil, and mild nausea, vomiting, and dyspepsia were also reported after oral lavender use (Koulivand et al., 2013 ▶). In addition, anticholinergic syndrome and supraventricular tachycardia were reported after lavender tea toxicity (Acikalin et al., 2012 ▶).

Small sample size and measurement tools were of the study limitations. We had to use a simple tool (VAS) to detect OD, because visiting patients was not reasonable due to the risk of the disease transmission. The follow up process was conducted by telephone, in our study. 

Another source of bias in this study is the lack of blinding. To eliminate this bias, patients in the control group could have taken a placebo syrup. 

Further studies with larger sample sizes, different control groups and standard measurement tools should be conducted. 

Although, OD significantly improved in both groups in the present study, but the amount of decrease in severity of OD was higher in the lavender group than the control group. Further studies with larger sample sizes and other measurement tools are suggested.

## Conflicts of interest

The authors have declared that there is no conflict of interest.
